# Low humidity enhances thermotolerance in *Caenorhabditis elegans*

**DOI:** 10.17912/micropub.biology.001404

**Published:** 2024-11-23

**Authors:** Michelle E. Brown, Diego A. Hernandez-Urbina, Caroline Kumsta

**Affiliations:** 1 Sanford Burnham Prebys Medical Discovery Institute, La Jolla, California, United States

## Abstract

Humidity is an important environmental factor that causes physiological changes in organisms. In humans, high humidity disrupts thermoregulation by limiting heat dissipation, leading to heat stress. While *Caenorhabditis elegans* lacks comparable thermoregulatory systems, humidity may still impact its heat tolerance by affecting cellular stress responses. We tested this by subjecting *C. elegans* to heat shock under different humidity conditions and found that lower humidity during heat shock improved survival compared to higher humidity. These findings demonstrate that humidity is an important variable affecting thermotolerance in *C. elegans* and should be standardized in heat-stress experiments.

**Figure 1. Low humidity improves C. elegans thermotolerance:**
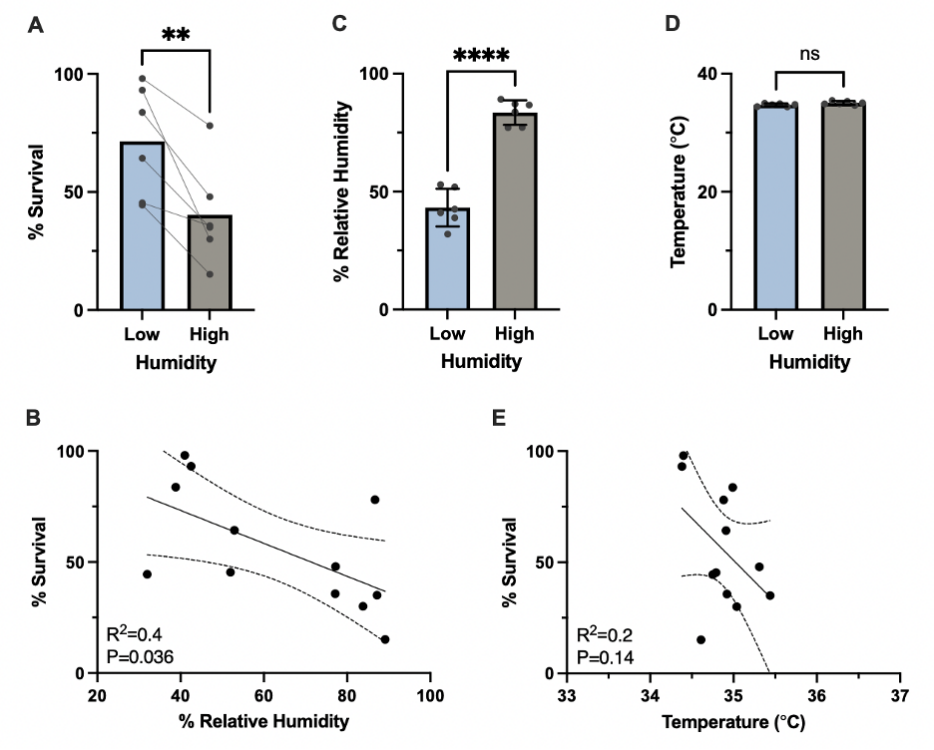
( **A** ) Percent survival of day-three adult wild-type animals in six paired thermorecovery assays (35°C for 6h) with N~100 animals per condition conducted at low (32-52%) and high (77-90%) relative humidity. Bars represent the mean survival rate. **P=0.0083 by paired *t* -test. ( **B** ) Survival rate plotted against relative humidity (%) for each experiment, with solid line for linear regression fit and dotted lines for 95% confidence intervals. P=0.036 by simple linear regression. ( **C** ) Mean relative humidity (%) recorded every 5 min during heat-shock exposure in each paired thermorecovery assay shown in (A). Mean +/- standard deviation, ****P<0.0001 by *t* -test. ( **D** ) Mean temperature (°C) recorded every 5 min during heat-shock exposure in each paired thermorecovery assay shown in (A). Mean +/- standard deviation, ns P=0.07 by *t* -test. ( **E** ) Survival rate plotted against mean temperature (°C) for each experiment, with solid line for linear regression fit and dotted lines for 95% confidence intervals. P=0.14 by simple linear regression.

## Description

Adaptation to environmental conditions, such as humidity, is critical for species survival and maintenance of homeostasis. Humidity can influence the physiological and behavioral responses in organisms of all kinds. For instance, many insects possess specialized hygroreceptors that detect moisture levels in their environment, which are important to direct reproduction behavior and survival (Bar-Zeev 1967) . On the other hand, some species are affected by humidity through physiological means rather than through specialized sensory mechanisms (Filingeri 2015) . This includes the nematode *C. elegans* , which is particularly vulnerable to both desiccation and overhydration, due to its cuticle's water permeability (Filingeri 2015; Russell et al. 2014) . While *C. elegans* is found in naturally humid environments (60-70% relative humidity, RH), such as compost and wooded areas (Indong et al. 2024; Schulenburg and Felix 2017) , it does not exhibit a strong preference for humidity under standard culture conditions (Russell et al. 2014) . However, when *C. elegans* is starved, its humidity preference becomes plastic and adapts to prior environmental exposure (Goodman and Sengupta 2019; Russell et al. 2014) .

Although *C. elegans* shows sensitivity and responsiveness to humidity, the broader impacts of humidity on its movement, behavior, survival, and overall health remain understudied. In humans, high humidity impairs thermoregulation by limiting the body's ability to cool itself through sweating, increasing susceptibility to heat stress and related illnesses (Davis et al. 2016) . This connection between humidity and heat stress in humans could suggest that humidity may similarly affect *C. elegans'* ability to tolerate heat, possibly by affecting water balance and other stress response mechanisms (Urso and Lamitina 2021) . To answer these questions, we sought to test whether humidity could directly influence *C. elegans* ' ability to tolerate heat stress. This potential influence of humidity on *C. elegans* ' heat shock response (HSR) raises concerns about observed and reported variability in heat-stress experiments (Golden et al. 2020) . Heat-stress experiments in *C. elegans* are performed in a variety of settings, ranging from water baths to incubators with varying humidity levels (Rubio-Tomas et al. 2024; Zevian and Yanowitz 2014) . Additionally, differences in regional climates and seasonal conditions could further introduce inconsistencies in experimental outcomes (Urban et al. 2021) .

To assess the impact of humidity on thermotolerance, we performed thermorecovery experiments of wild-type *C. elegans* on day three of adulthood by subjecting them to a six-hour heat shock at ~35°C with variable relative humidity conditions in two Heratherm heat-shock incubators. We chose day-three adults for these assays as they are more consistent in their thermotolerance responses compared to day-one adults, likely due to the reported decline in the HSR and HSF-1 activity at the onset of reproductive maturity on day one (Cohen et al. 2010; Labbadia and Morimoto 2015) . To achieve high-humidity conditions (77-90% RH), two water troughs containing 400 mL of water were placed into the heat-shock incubator, while the low-humidity conditions (32-52% RH) were maintained in the second heat-shock incubator by omitting the water troughs. We found significantly higher survival in worms exposed to lower humidity levels (32-52% RH) during heat shock compared to those subjected to higher humidity levels (77-90% RH) ( **Fig. 1A** ), and a negative linear relationship between humidity and survival rate (R ^2^ =0.4, P=0.036, linear regression) ( **Fig. 1B** ). Each percentage point increase in humidity resulted in a 0.7% decrease in survival, demonstrating that increased humidity negatively impacted thermotolerance. The mean relative humidity in the two incubators was statistically different from each other (P<0.0001, *t* -test across six experiments) ( **Fig. 1C** ), and humidity levels remained stable over the duration of each six-hour heat shock. Importantly, the average temperatures during the six-hour heat shock were not significantly different between the two humidity conditions (P>0.05, *t* -test across six experiments) ( **Fig. 1D** ), indicating that temperature differences did not account for the observed survival variation. Additionally, there was no significant correlation between temperature fluctuations and survival rates (R ^2^ =0.2, P>0.05, linear regression), further confirming that the observed effects on survival were due to humidity rather than temperature variability ( **Fig. 1E** ).

In summary, our findings indicate that lower humidity during heat shock enhances *C. elegans* survival, highlighting humidity as a critical factor in heat-stress resilience. Since the animals were maintained at high humidity levels (~74% RH) before the heat shock, the low-humidity conditions (32-52% RH) during heat stress may have been sufficiently low to induce water loss through the cuticle, leading to osmotic stress, potentially activating the hypertonic stress response (HTSR) (Lamitina et al. 2004) . While the HTSR leads to genetic adaptations that protect *C. elegans* from osmotic stress, there is potential for hormetic cross-protection between hypertonic stress and heat stress via the upregulation of SKN-1/Nrf2 (Dues et al. 2016; Urso et al. 2020) , which may increase overall stress resistance. Severe hypertonic stress can induce endogenous protein aggregation (Burkewitz et al. 2011) , similar to heat-shock conditions, and can lead to the formation of HSF-1-containing nuclear stress bodies (Deonarine et al. 2021) . The combination of heat stress with potential water loss due to low-humidity conditions may thus cause additive activation of HSF-1. Since increased levels of HSF-1 improve the survival of *C. elegans* in thermotolerance experiments (Hsu et al. 2003; Morley and Morimoto 2004; Kumsta et al. 2017) , such additive activation could explain the enhanced heat-stress survival observed under low-humidity conditions. In contrast, the higher humidity range (77-90% RH), being slightly higher than our lab’s standard maintenance humidity (~74%), may have caused increased water uptake in *C. elegans* , potentially leading to overhydration. This could limit the activation of protective pathways, like the HTSR, and impair the worms' capacity to maintain cellular homeostasis, contributing to decreased survival under heat stress. It would be interesting to investigate the activation of specific stress response pathways, such as the HTSR and HSR, under different humidity conditions. Additionally, it remains to be investigated how the temporal activity of HSF-1 throughout adulthood could affect the HSR under varying humidity conditions. Extricating the dynamics of these pathways with age and upon varied humidity conditions may contribute to a better understanding of heat-stress resistance. Moreover, it would be valuable to test whether humidity influences resistance to other environmental stressors, such as oxidative stress, to determine if the effects of humidity on thermotolerance are part of a broader pattern of stress response regulation.

While our method of controlling humidity levels via inclusion of water troughs effectively created distinct humidity levels and demonstrated that low humidity improves *C. elegans* survival upon heat stress, there was some variability between experimental replicates. A more precise, automated system for controlling humidity could reduce this variability and enable testing of additional humidity levels in future experiments.

Taken together, this study highlights the importance of controlling humidity in *C. elegans* heat-stress experiments. Variability in thermotolerance assays has been previously reported (Golden et al. 2020) , with differences in environmental conditions, such as humidity, possibly contributing to inconsistencies across studies. Accurate humidity control could ameliorate these discrepancies between labs and improve reproducibility. Recording humidity levels could be a first step toward standardizing conditions, which could increase reliability, and provide clearer insights into the mechanisms of the HSR. Given the evolutionary conservation of stress response pathways, these findings may offer broader implications for understanding how humidity affects thermotolerance across diverse species, potentially informing studies on environmental-stress adaptation and resilience.

## Methods

*Strains*

Wild-type *C. elegans* (N2) were maintained on *E. coli* OP50-seeded nematode growth medium (NGM) plates at 20°C and approximately 74% relative humidity (RH). Humidity in the maintenance incubator was measured every five minutes by TraceableGO Bluetooth Datalogging Hygrometer (Fisherbrand, #15079679).

*Thermorecovery assay*

Approximately 50 eggs were transferred from an N2 maintenance plate onto four 6 cm OP50-seeded NGM plates, for a total of 200 worms per experiment. Worms were grown to day two of adulthood at 20°C and ~74% RH and then transferred to fresh OP50-seeded NGM plates to separate them from progeny. On day three of adulthood, two plates (totaling 100 animals) were placed in a Heratherm (Thermo Scientific) incubator set to ~35°C and ~84% RH for six hours. In parallel, the remaining two plates were placed in a Heratherm (Thermo Scientific, #51028065H) incubator set to ~35°C and ~43% RH for six hours. Humidity was manipulated by adding or removing two 11x13 cm water troughs from the respective incubators with 400 mL H _2_ O in each trough at least 12 hours prior to the heat-shock experiment. Humidity and temperature were recorded every 5 min using a TraceableGO Bluetooth Datalogging Hygrometer (Fisherbrand, #15079679) and exported to Excel. After the six-hour heat shock, the animals were returned to the maintenance incubator set to 20°C and ~74% RH for a 24-hour recovery period. Survival of the animals was scored by gently tapping the plates and prodding the worms’ tails with a platinum wire. Animals exhibiting crawling or lateral head movement were scored as alive, while those with no movement were scored as dead. Average survival was calculated per 100 worms. For statistical analyses, two-tailed paired and unpaired *t* -tests, and linear regression were performed using GraphPad Prism 10.3.1.

## Reagents

The wild-type *Caenorhabditis elegans* ( N2 ), is available from the *Caenorhabditis* Genetics Center.

Hygrometer; Fisherbrand TraceableGO Bluetooth Datalogging Hygrometer #15079679

Heat-shock incubators; Thermo Scientific Heratherm 194L GP Incubator #51028065
